# Voxel-Mirrored Homotopic Connectivity Associated With Change of Cognitive Function in Chronic Pontine Stroke

**DOI:** 10.3389/fnagi.2021.621767

**Published:** 2021-02-18

**Authors:** Luobing Wu, Caihong Wang, Jingchun Liu, Jun Guo, Ying Wei, Kaiyu Wang, Peifang Miao, Yingying Wang, Jingliang Cheng

**Affiliations:** ^1^Henan Key Laboratory of Magnetic Resonance Function and Molecular Imaging, Department of MRI, The First Affiliated Hospital of Zhengzhou University, Zhengzhou, China; ^2^Tianjin Key Laboratory of Functional Imaging, Department of Radiology, Tianjin Medical University General Hospital, Tianjin, China; ^3^Department of Radiology, Tianjin Huanhu Hospital, Tianjin, China; ^4^GE Healthcare MR Research, Beijing, China

**Keywords:** VMHC, fMRI, cognitive function, pontine stroke, right hemisphere

## Abstract

Recent neuroimaging studies have shown the possibility of cognitive impairment after pontine stroke. In this study, we aimed to use voxel-mirrored homotopic connectivity (VMHC) to investigate changes in the cognitive function in chronic pontine stroke. Functional MRI (fMRI) and behavioral assessments of cognitive function were obtained from 56 patients with chronic pontine ischemic stroke [28 patients with left-sided pontine stroke (LP) and 28 patients with right-sided pontine stroke (RP)] and 35 matched healthy controls (HC). The one-way ANOVA test was performed for the three groups after the VMHC analysis. Results showed that there were significant decreases in the bilateral lingual gyrus (Lingual_L and Lingual_R) and the left precuneus (Precuneus_L) in patients with chronic pontine ischemic stroke compared to HCs. However, in a *post-hoc* multiple comparison test, this difference remained only between the HC and RP groups. Moreover, we explored the relationship between the decreased *z*-values in VMHC and the behavior-task scores using a Pearson's correlation test and found that both scores of short-term memory and long-term memory in the Rey Auditory Verbal Learning Test were positively correlated with *z*-values of the left lingual gyrus (Lingual_L), the right lingual gyrus (Lingual_R), and the left precuneus (Precuneus_L) in VMHC. Besides that, the *z*-values of Precuneus_L in VMHC were also negatively correlated with the reaction time for correct responses in the Flanker task and the spatial memory task. In conclusion, first, the lingual gyrus played an important role in verbal memory. Second, the precuneus influenced the working memory, both auditory-verbal memory and visual memory. Third, the right-sided stroke played a greater role in the results of this study. This study provides a basis for further elucidation of the characteristics and mechanisms of cognitive impairment after pontine stroke.

## Introduction

Cognitive impairment is common and persistent among long-term stroke survivors (Nys et al., 2007; Delavaran et al., 2017; Groeneveld et al., 2019). This important clinical manifestation is not only present in patients with cortical stroke but also in the subcortical brain regions of patients with pontine stroke (Maeshima et al., 2012). Moreover, most patients with pontine stroke still leave varying degrees of cognitive impairment during follow-up (Wei et al., 2020). In fact, cognitive impairment could exert a further negative influence on the daily lives of patients, including deficits in memory and recall, the response capacity to handle complex activities, and the ability to handle the challenges of life (Shimada et al., 2016; Braga et al., 2018; Gajewski et al., 2018), and they deserve more attention.

Characteristics of neural mechanisms are known to be linked with clinical outcomes in patients with stroke, neuroimaging studies like resting-state functional connectivity have provided new insights into functional impairment and improvement. Previous studies have shown that disturbed functional connectivity associated with cognitive function could occur immediately after stroke, and this alteration may persist for a long time (Golestani et al., 2013; Liu et al., 2016). Chen et al. (2019) reported that patients with pontine stroke might exhibit more severe cognitive damage, especially memory processing, compared to patients with subcortical stroke as evidenced by different patterns of functional connectivity alterations in the chronic phase. Multimodal MRI studies have also found that pontine stroke poses a potential risk of memory impairment using dynamic functional network connectivity (Wang et al., 2020) or structural covariance networks (Wei et al., 2020). However, the neurological mechanisms of stroke-induced cognitive impairment in patients with pontine stroke remain to be elucidated, and not only the memory function.

Homotopic cortical areas in both hemispheres play a crucial role in neuroplasticity and in the reorganization of the brain. Impaired interhemispheric functional coordination in the brain regions that are involved in the clinical characteristics and impairment of cognitive performance has been reported in neuroimaging studies (Yang et al., 2017; Fan et al., 2018). As one such approach that reflects interhemispheric homotopic coordination by integrating brain functions underlying coherent cognition, emotion, and behavior control (Zuo and Xing, 2014; Fan et al., 2018), voxel-mirrored homotopic connectivity (VMHC) has been considered a reliable approach that may help us explore and recognize the potential role of behavior-associated alterations of interhemispheric functional connectivity. A considerable number of studies have focused on how damage in VMHC influences stroke (Tang et al., 2016; Shan et al., 2018; Chen et al., 2021). In view of a previous study which demonstrated that connectivity between homotopic FC was significantly associated with clinical performance of the motor control (Urbin et al., 2014), previous VMHC reports (Shan et al., 2018) have only focused on the potential mechanisms of motor function in chronic pontine stroke, but little is known about the relatively hidden mechanism of cognitive impairment. However, cross-hemispheric functional connectivity of ROI to ROI has also revealed that interhemispheric connections involving homotopic areas have the highest degree of efficiency in spatial cognitive impairments after stroke (Ptak et al., 2020). Hence, we speculated whether such patterns of memory processing and spatial cognitive impairments are seen in patients with chronic pontine stroke with the use of VMHC.

To address the aforementioned problems, driven by the interest in cognitive changes of patients with pontine stroke, we used VMHC to explore the difference between pontine stroke patients and healthy controls (HCs). More focus should be given to the following two points of this study. First, we chose to predict the resting-state brain activity in the chronic stage of stroke because couplings between resting-state functional connectivity and individual's task performance or behavior change dynamically with stroke progression of an individual and the strength of couplings increases as the recovery from stroke progresses (Hu et al., 2017). The other reason for choosing the chronic stage was to minimize the impact of motor function on cognitive function, considering that the recovery of motor function reached a stable state after stroke (Kwakkel and Kollen, 2013). Second, different lesion locations would characterize individual contribution to functional outcomes depending on the side of the hemispheres (Cheng et al., 2014). In addition, differences between left and right lesions have been revealed in previous studies (Jiang et al., 2017; Wang et al., 2019c), so patients were subdivided into two groups: a left stroke group and a right stroke group.

Therefore, in this study we aimed to: (1) verify the changes in brain function as measured by VMHC after chronic pontine stroke; (2) identify the lesion-side effect of the alterations of VMHC in patients with pontine stroke; and (3) assess the correlation between the significantly different brain regions and assessments of cognition.

## Materials and Methods

### Participants

Patients with chronic pontine stroke and healthy subjects were recruited from The First Affiliated Hospital of Zhengzhou University, Tianjin Medical University General Hospital, and Tianjin Huanhu Hospital. The experimental protocol was approved by the local medical research ethics committee, and written informed consent was obtained from all participants. In order to ensure the quality of the data, the three cooperative hospitals jointly drafted a strict protocol that formulated and unified the enrollment standards and scanning specifications, organized personnel training, and coordinated the scanning parameters with General Electric (GE) engineers. At the same time, as much as possible, the cognitive test adopted machine evaluation assessment without subjective color. The inclusion criteria for patients with stroke were as follows: (a) the first-onset of stroke with a single lesion that occurred in the pontine area and left and right lesions could be clearly distinguished; (b) the observation time after stroke onset was > 6 months to ensure that the patients were at a stable chronic stage; and (c) right-handed patients aged 40–80 years. The exclusion criteria were as follows: (a) a previous history of stroke; (b) bilateral or multiple lesions; (c) excessive white matter demyelination, with a modified Fazekas scale for white matter hyperintensities > 1 (Fazekas et al., 1987); (d) a history of mental illness; (e) craniocerebral trauma or other physical organic lesions; and (f) any contraindications to MRI examination. Then, the enrolled patients who met the criteria were subdivided into a left-sided pontine stroke (LP) group and a right-sided pontine stroke (RP) group according to the location of the lesion. The recruitment requirements of the normal HC group were as follows: (a) gender, age, and education level matched with those of the patient group; (b) no cognitive or physical dysfunction; and (c) the exclusion criteria listed above as for patients with pontine stroke. A total of 91 right-handed participants (35 female; mean age, 57.1 ± 7.3 years; 28 LP, 28 RP), and 35 HCs (13 female; mean age, 55.7 ± 7.0 years) were included.

### Behavioral Assessment

The patients were assessed and the Fugl–Meyer Assessment (whole extremity, total 100 scores) scores were recorded. All patients and normal volunteers who met the above criteria underwent behavioral assessments before and after the collection of MRI images, including: (a) the Rey Auditory Verbal Learning Test (RAVLT) for evaluating verbal short-term memory (VSTM) and verbal long-term memory (VLTM). The total number of correctly recalled words was recorded as the two terms of RAVLT scores; (b) the Flanker task and the spatial memory task. The selective attention tasks were employed to assess the ability of visual attention, interference suppression, and motor responses of an individual. The details have been previously reported (Shimada et al., 2016; Gajewski et al., 2018). The accuracy rate (ACC, equal to the ratio of the number of correct responses and the total number of possible correct responses) and the average reaction time (RT) for correct responses (time between presentation and manual response) were taken as the dependent variables for the above two spatial tasks.

The evaluation work was supervised by two clinicians with rich clinical experience, who jointly determined the reliability of the results after unifying the standards.

### MRI Data Acquisition

All MRI data were obtained using a GE Discovery MR750 3.0T MRI scanner (GE Medical Systems, Waukesha, WI). The imaging parameters for the three hospitals were consistent. These were: (1) resting-state functional MRI (fMRI) with an echo-planar (EPI) sequence: repetition time/echo time (TR/TE) = 2,000/30, fractional anisotropy (FA) = 90°, field-of-view (FOV) = 240 × 240 mm, matrix = 220 × 220, slice thickness = 4 mm, gap = 0.5 mm, interleaved transversal slices = 32, time-point = 180 and (2) high-resolution 3D-T1-weighted structural images with magnetization prepared rapid gradient echo (MPRAGE) sequence: TR/TE = 8.2/3.2, FA = 12°, FOV = 256 × 256 mm, matrix = 256 × 256, slice thickness = 1 mm. During the process of image acquisition, participants were instructed to lie flat on the examination bed, put noise-attenuating earplugs into both ears to reduce the noise during the scan, and place their head firmly between sponge pads on the left and right sides of the head to keep the head in place and to further reduce noise exposure. At the same time, participants were told to stay awake, breathe smoothly, try not to make any movements, or think intentionally.

**Figure 1:**
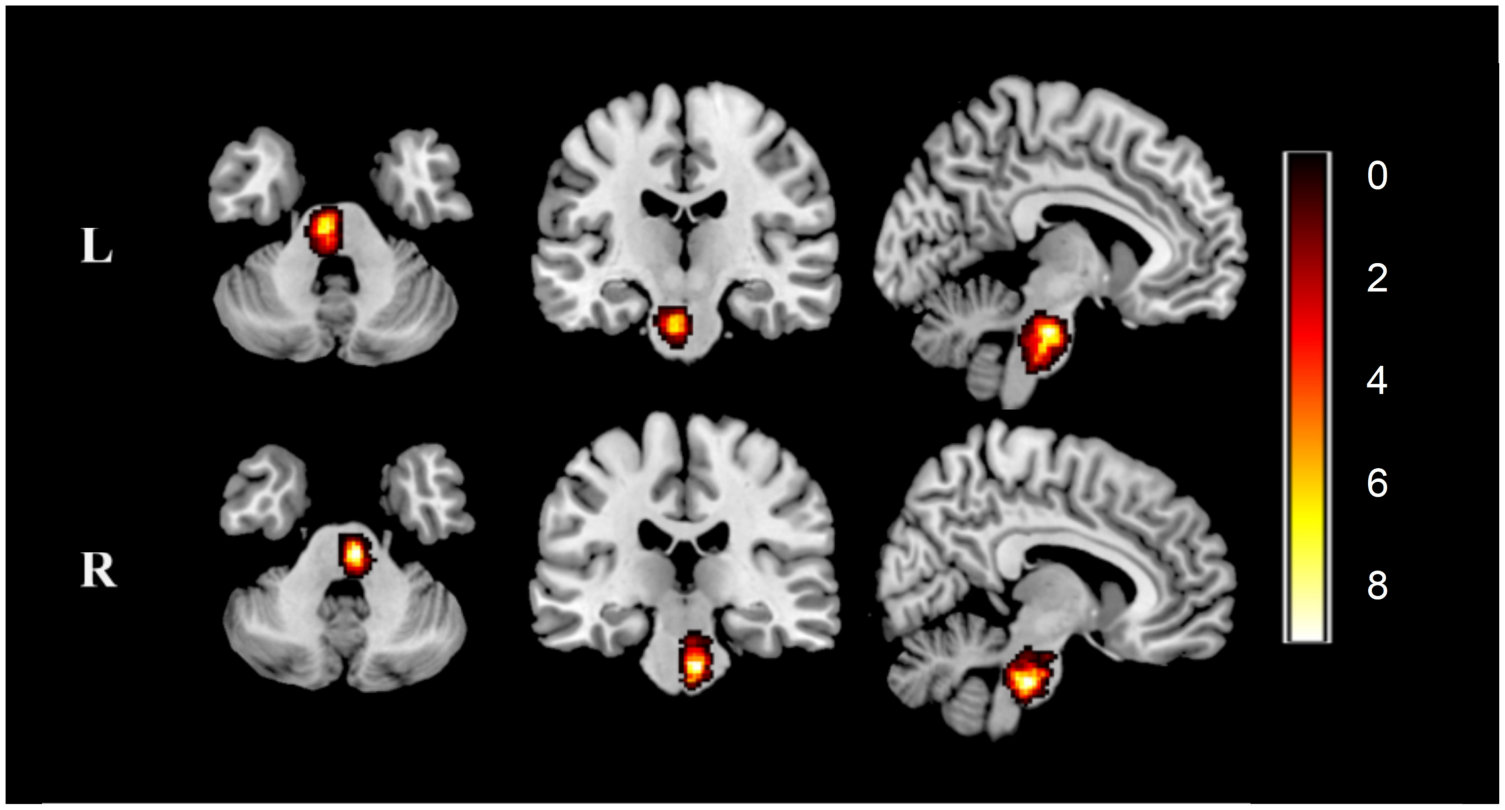
Lesion overlap map across stroke patients. Lesion maps were normalized to an MNI reference brain. Color bar indicates the number of patients with stroke having lesions in each voxel. L, left-sided pontine stroke (*n* = 28); R, left-sided pontine stroke (*n* = 26); MNI, Montreal Neurological Institute.

We manually outlined the lesion profiles on high-resolution 3D-T1-weighted MRI images slice by slice on MRIcron (http://www.mccauslandcenter.sc.edu/mricro/mricron). The generated lesion masks along with the original 3D-T1 images were normalized to the Montreal Neurological Institute (MNI) space for each patient. Next, all normalized patient lesion masks were overlapping by calculation. Finally, the individual lesion masks were averaged and overlaid with a template to create the lesion overlap map, as shown in Figure 1.

### MRI Data Processing

The preprocessing of the resting-state fMRI data were performed on the software Data Processing Assistant for Resting-State fMRI (DPARSF, http://resting-fmri.sourceforge.net) with Statistical Parametric Mapping (SPM8, http://www.fil.ion.ucl.ac.uk/spm). Procedures were as follows: (1) the first 10 time points were discarded considering the magnetization balance and the subject's adaptation to the environment; (2) time correction for acquisition delay of slices; (3) realignment for head motion correction, in which no participant was excluded due to excessive head movement (rotation, translation, or head movement > 2° or 2 mm). Moreover, mean framewise displacement (FD) was calculated by averaging the FD of each participant across the time points, and three patients were excluded owing to a mean FD > 0.5, one from HC and two from RP. We also found no significant differences in mean FD among the groups (*P* = 0.800); (4) coregistration of each T1 image to the individual functional image after motion correction, and then segmentation into gray matter, white matter, and cerebrospinal fluid; (5) normalization of the functional images to the MNI space and then re-sampling into a voxel size of 3 × 3 × 3 mm; (6) removal of nuisance covariates (cerebrospinal fluid, white matter, 6-head motion parameters, 6-head motion parameters at one time point earlier, and the 12 corresponding squared items (Friston 24-parameter model) as covariates) from the data by linear regression; (7) spatial smoothing with a 6-mm full-width-at-half-maximum (FWHM) Gaussian kernel; and (8) temporal bandpass filtering (0.01–0.08 Hz) and linear detrending.

After preprocessing, the individual VMHC maps were calculated. Specifically, we calculated the Pearson's correlation coefficient between the residual time series of each voxel and that of its symmetrical interhemispheric counterpart. Correlation values were then Fisher *Z*-transformed, and *z*-maps were obtained last to increase the distribution normality. The resultant values (*z*-values) were referred to as the VMHC and were used for subsequent group-level analyses (Zuo et al., 2010).

### Statistical Analysis

A two-sample *t*-test was used to compare demographic and clinical characteristics between the LP and the RP groups except for the categorical data, including the duration time after stroke, the lesion volume, and the Fugl–Meyer Assessment (FMA) score. The one-way ANOVA was used to compare age, education level, and assessments of cognitive variables among the HC, LP, and RP groups. Gender was analyzed using a case-weighted chi-square test as categorical data. With individual age, gender, education level, mean FD, and lesion volumes as covariates, one-way ANOVA analysis was also performed to identify the differences of VMHC maps among different groups based on the Gaussian Random Field theory with a cluster-level family-wise error (FWE) correction (*P* < 0.05), accompanied by pairwise comparisons of *post-hoc* multiple corrections (Scheffe). The VMHC values of the brain regions showing abnormal interhemispheric connectivity were then normalized, extracted, and calculated for correlation with the scores of the clinical tasks using the Pearson's correlation coefficient. Statistical Product and Service Solutions (SPSS) version 23.0 statistical software (IBM Corporation, Armonk, NY, USA) was used to compare clinical measurements and correlation analyses.

## Results

### Sample Characteristics

The final population included 54 patients and 34 HCs. Among the 54 patients, there was a mean duration after stroke of 12.5 ± 7.5 months, a mean lesion volume of 0.39 ± 0.43 cc^3^, and a mean FMA score of 94.9 ± 11.5. No significant differences were found for demographic and clinical characteristics between the LP and RP groups. There were no significant differences in gender (χ^2^ = 1.895, *P* = 0.169), age (*P* = 0.386), and education level (*P* = 0.899) between the two stroke groups. There were no significant differences in gender (χ^2^ = 2.350, *P* = 0.125), age (*P* = 0.225), and education level (*P* = 0.359) among the groups. As for the clinical assessment scores, no significant differences were found, except for the ACC of the spatial memory task (*P* = 0.039). More information is displayed in Table 1.

**Table 1:** Demographic and clinical characteristics.

	**Left-sided stroke (*n =* 28)**	**Right-sided stroke (*n =* 26)**	***P*-value/t (LP vs. RP)**	**Healthy Controls (*n =* 34)**	***P*-value/F (ANOVA)**
Gender(male/female)	18/10	14/12	0.169(χ^2^)	21/13	0.125(χ^2^)
Age(year)	58.7 ± 6.6 (49–78)	56.9 ± 8.3 (42–72)	0.386	55.4 ± 7.0 (44–75)	0.225
Education(year)	10.2 ± 3.8 (0–16)	10.3 ± 3.8 (22)	0.899	11.4 ± 3.0 (6–16)	0.359
Mean *FD*	0.14 ± 0.10 (0.05–0.43)	0.14 ± 0.08 (0.03–0.32)	0.603	0.14 ± 0.07 (0.04–0.38)	0.800
Duration(month)	11.9 ± 6.6 (6–28)	13.1 ± 8.5 (6–35)	0.574	…	…
Lesion volume(cc^3^)	0.46 ± 0.53 (0.04–2.48)	0.31 ± 0.28 (0.01–1.16)	0.182	…	…
*FMA*	95.5 ± 13.4 (30–100)	94.3 ± 9.1 (65–100)	0.700	…	…
***RAVLT***					
VSTM	42.1 ± 10.4 (22–65)	45.0 ± 13.5 (26–73)	0.391	48.8 ± 9.0 (30–71)	0.054
VLTM	10.4 ± 2.7 (4–15)	9.3 ± 3.3 (2–15)	0.238	10.9 ± 3.2 (0–15)	0.163
***Flanker task***					
ACC	0.94 ± 0.10	0.97 ± 0.04	0.225	0.97 ± 0.05	0.211
RT(msec)	696.20 ± 215.55	693.43 ± 230.20	0.968	697.04 ± 232.54	0.998
***Spatial memory task***					
ACC	0.85 ± 0.16	0.90 ± 0.06	0.107	0.92 ± 0.08	0.039^*^
RT(msec)	937.23 ± 265.61	946.96 ± 229.66	0.890	904.97 ± 206.89	0.773

Data represent as mean ± SD (minimum-maximum);

* P < 0.05.

*mean FD, mean frame-wise displacement (FD); FMA, the Fugl–Meyer Assessment (whole extremity, total 100 scores); RAVLT, the Rey Auditory Verbal Learning Test; VSTM, verbal short-term memory; VLTM, verbal long-term memory; ACC, accuracy rate; RT, response time for correct response*.

### Difference in VMHC Among Groups

**Figure 2:**
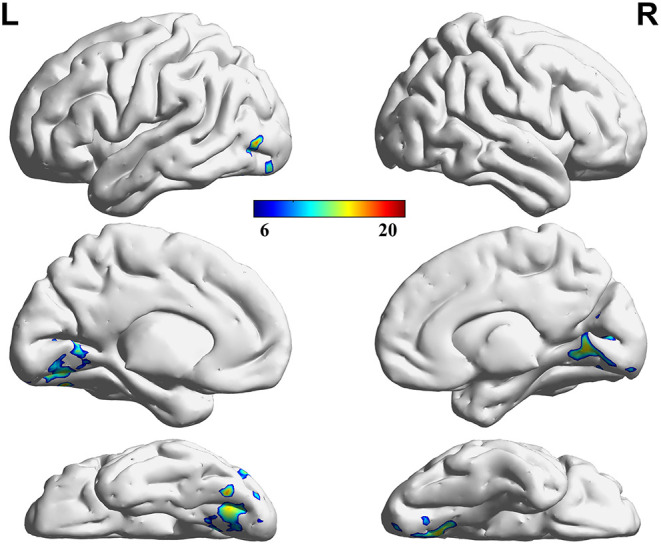
Regions showing significant differences in VMHC among groups. The blue to red color bar indicates the F-value from the one-way ANOVA analysis among groups. Comparisons were corrected by using the Gaussian Random Field theory with a cluster-level family-wise error (FWE) correction (*p* < 0.05).

**Figure 3:**
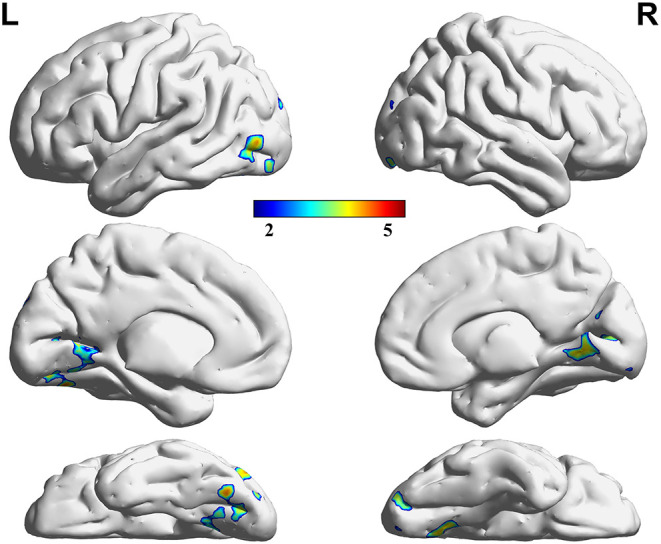
Regions showing significant differences in VMHC between the HC and RP groups. The blue to red color bar indicates the *t*-value from the *post-hoc* multiple correction test (Scheffe) between the HC and RP groups (*p* < 0.05).

Three decreased VMHC values were found in the comparisons among the HC, LP, and RP groups. These were the left lingual gyrus (Lingual_L), the right lingual gyrus (Lingual_R), and the left precuneus (Precuneus_L). The same significant differences were found between the HC and RP groups on the *post-hoc* multiple comparison test. There were no significant differences in VMHC intensity in the comparison between the LP and the RP groups or the HC and the LP groups. These details are shown in Table 2, Figures 2, 3.

**Table 2:** Results of VMHC among groups and the *post-hoc* multiple comparison test.

**Item**	**Peak Region(*AAL*)**	**Peak coordinates** ***MNI***			**Cluster size (voxels)**	**Peak Intensity**
		**x**	**y**	**z**		
***F***	Lingual_L	−33	−69	−18	352	15.80
	Lingual_R	24	−81	21	312	15.11
	Precuneus_L	−21	−78	21	83	17.49
***HC vs. RP***	Lingual_L	−33	−69	−18	424	4.60
	Lingual_R	24	−81	21	376	4.79
	Precuneus_L	−21	−78	21	162	5.11

*VMHC, voxel-mirrored homotopic connectivity; HC, healthy controls; RP, right-sided stroke; F, one-way ANOVA analysis among groups; HC vs. RP, the post-hoc multiple comparison test between the HC and RP groups; AAL, Anatomical Automatic Labeling; MNI, Montreal Neurological Institute; Lingual_L, left lingual gyrus; Lingual_R, right lingual gyrus; Precuneus_L, left precuneus*.

### Correlational Analysis

**Figure 4:**
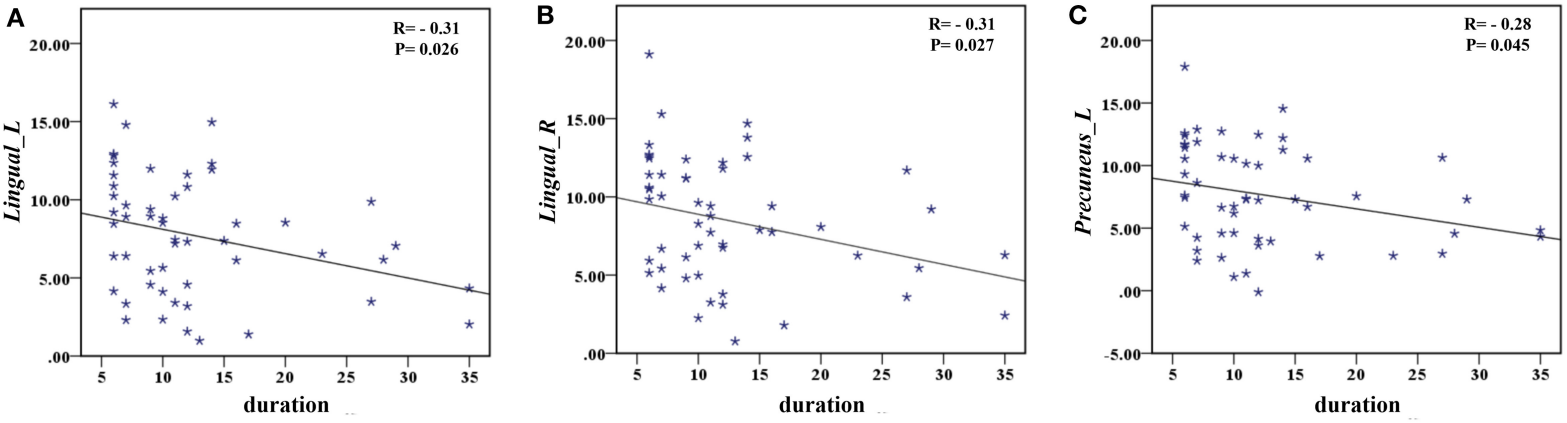
**(A)** Correlation between *z*-values of Lingual_L in VMHC and the duration since stroke. **(B)** Correlation between *z*-values of Lingual_R in VMHC and the duration since stroke. **(C)** Correlation between *z*-values of Precuneus_L in VMHC and the duration since stroke.

**Figure 5:**
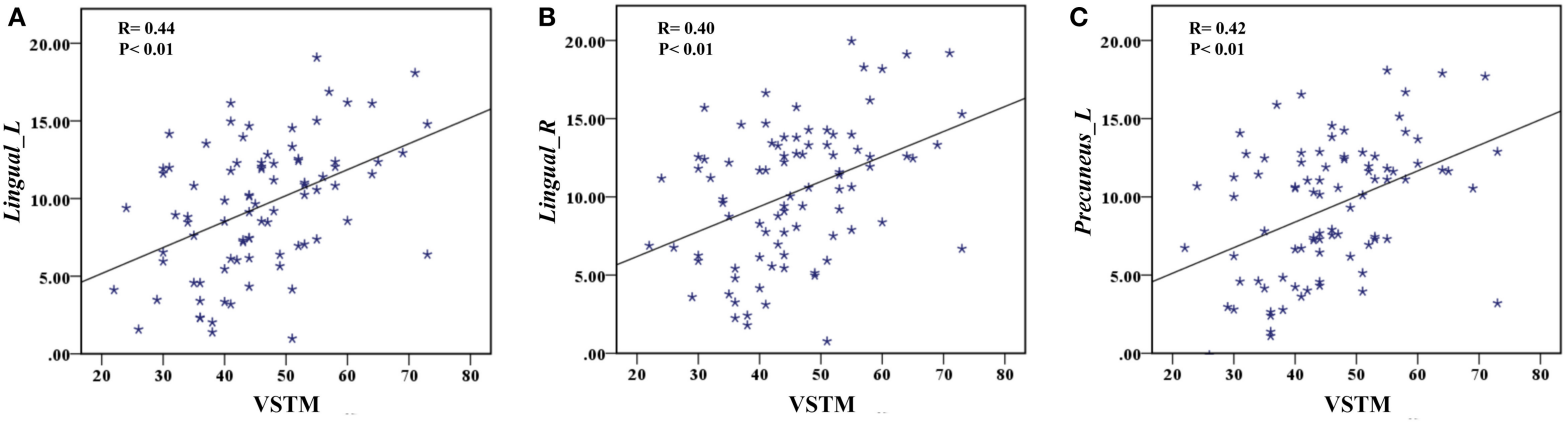
**(A)** Correlation between *z*-values of Lingual_L in VMHC and the VSTM scores. **(B)** Correlation between *z*-values of Lingual_R in VMHC and the VSTM scores. **(C)** Correlation between *z*-values of Precuneus_L in VMHC and the VSTM scores.

**Figure 6:**
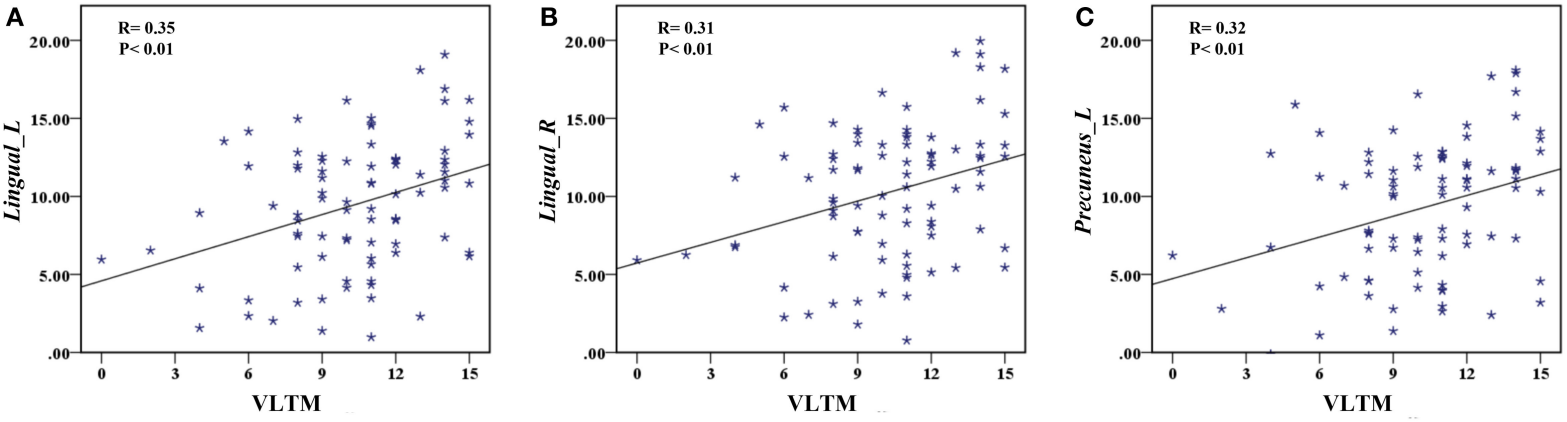
**(A)** Correlation between *z*-values of Lingual_L in VMHC and the VLTM scores. **(B)** Correlation between *z*-values of Lingual_R in VMHC and the VLTM scores. **(C)** Correlation between *z*-values of Precuneus_L in VMHC and the VLTM scores.

**Figure 7:**
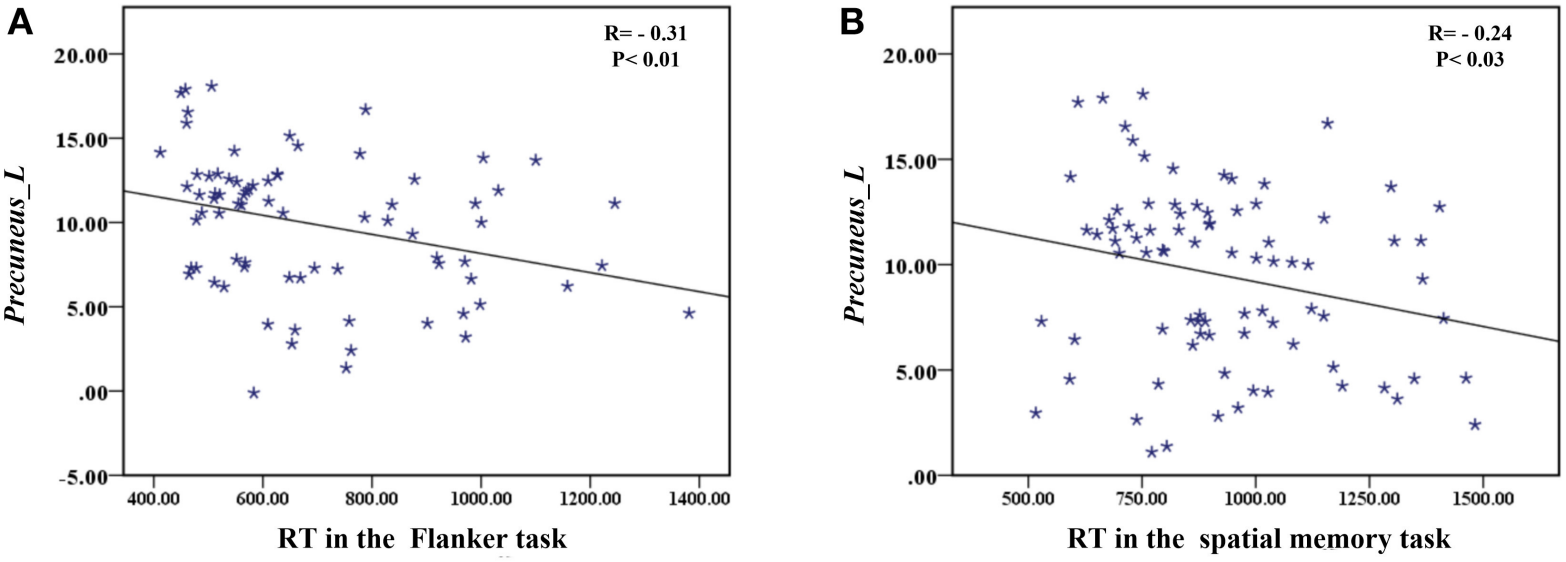
**(A)** Correlation between *z*-values of Precuneus_L in VMHC and the RT for correct responses in the Flanker task. **(B)** Correlation between *z*-values of Precuneus_L in VMHC and the RT for correct responses in the spatial memory task.

The correlation analysis showed that the duration since stroke was negatively correlated with the *z*-values of the Lingual_L, Lingual_R, and Precuneus_L in VMHC (*r* = −0.31, *r* = −0.31, and *r* = −0.28, respectively) (Figure 4). As for cognitive assessments, both the VSTM and VLTM scores were positively correlated with the *z*-values of the Lingual_L, Lingual_R, and Precuneus_L in VMHC (*r* = 0.44, *r* = 0.40, and *r* = 0.42, respectively; *r* = 0.35, *r* = 0.31, and *r* = 0.32, respectively) (Figures 5, 6). The correlation between the VSTM scores and the decreased *z*-values in VMHC was the strongest. In addition, there was a negative correlation between the *z*-values of the Precuneus_L in VMHC and the RTs for correct responses in the Flanker task (*r* = −0.31, *P* < 0.01) and in the spatial memory task (*r* = −0.24, *P* < 0.03) (Figure 7).

## Discussion

In this study, we have explored the change of VMHC with patients with chronic pontine stroke and the potential relationship between the change of VMHC and the behavior-task scores. Our main novel findings were as follows: (1) compared with HCs, the VMHC of the bilateral lingual gyrus and the Precuneus_L were significantly decreased in patients with stroke, and these alterations would get more obvious with prolonged duration since chronic stroke; (2) compared with the LP group, the RP group showed greater abnormalities of VMHC in the results; and (3) the decreased *z*-values of the bilateral lingual gyrus in VMHC predicted poor RAVLT scores, the decreased *z*-values of the Precuneus_L in VMHC predicted not only the poor RAVLT scores but also the prolonged RTs for correct responses in the spatial tasks. Collectively, these findings indicated that the VMHC could provide neurological information to forecast cognitive performance.

The VMHC of the patients was significantly reduced in the occipital cortex away from the pontine. The current result might suggest that pontine stroke has a greater effect on global function than on the local function of the brain, which is consistent with the previous finding that brainstem stroke significantly attenuates long-range functional connectivity (Salvador et al., 2005). Some studies infer that the mechanisms underlying the deficits in VMHC could be related to widespread white matter-integrity abnormalities, dysfunctions in local gray matter structure, and the reorganized pattern of pathways (Yuan et al., 2012; Ding et al., 2015). Therefore, the underlying reason for the change of VMHC is most likely because of the special anatomical structure of the pontine, which involves a large number of ascending and descending fibers staggered throughout the pontine (Querol-Pascual, 2010), and the pontine nucleus works as a relay station for transmitting information from the cerebral cortex. Since structural damages of patients with pontine stroke exist (Jiang et al., 2017; Guo et al., 2019), it can lead to long-range cortical functional damage.

**Figure 8:**
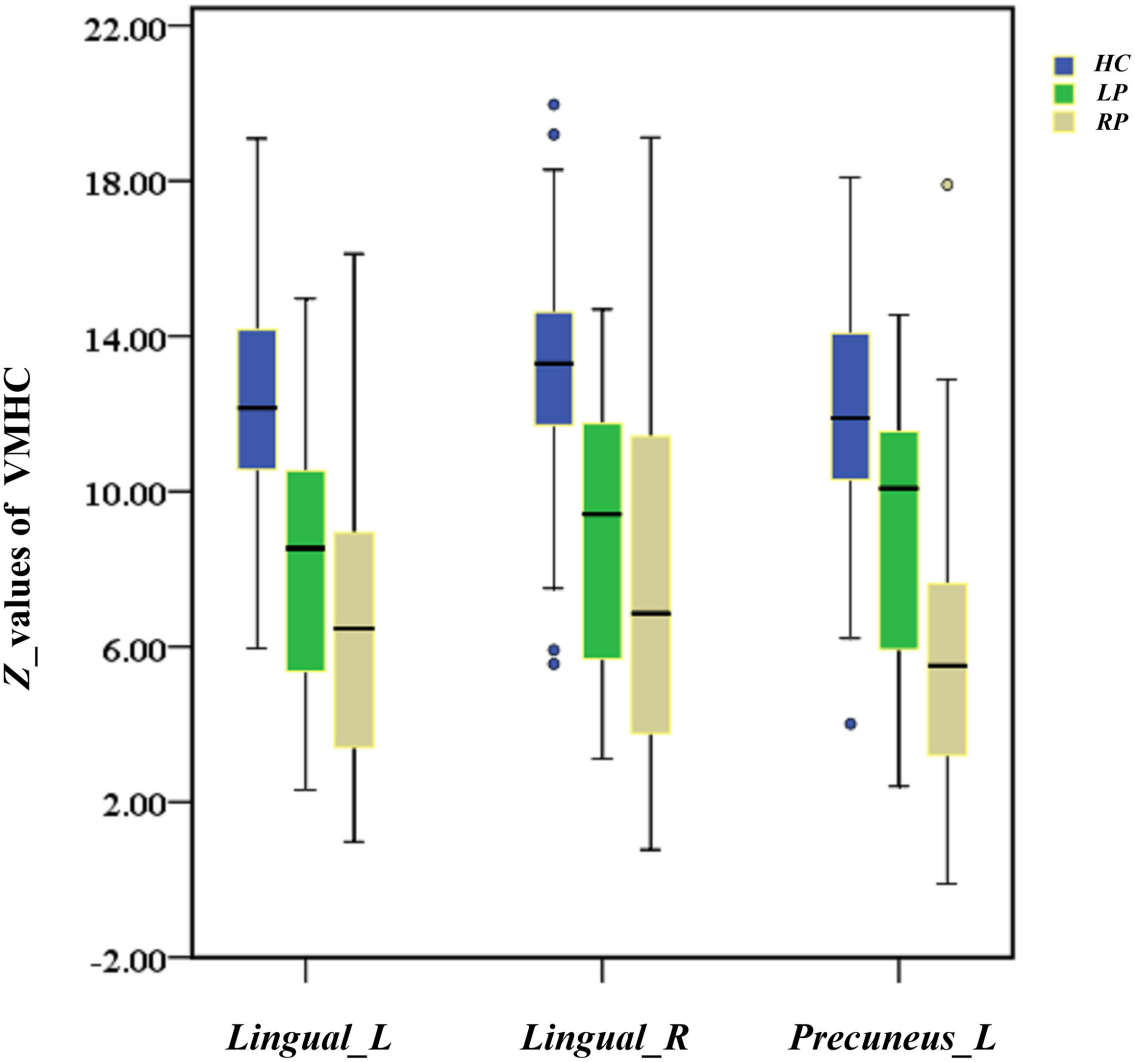
The box diagram shows the distribution of *z*-values of Lingual_L, Lingual_R, and Precuneus_L in VMHC among the groups (HC, LP, RP). Blue for HC, healthy controls; green for the LP, left-sided stroke; yellow for the RP, right-sided stroke.

A significant finding of this study was that the RP group exhibited a more extensive VMHC decrease than the LP group in a *post-hoc* multiple comparison test, although these two groups of patients did not differ in terms of any demographic or clinical characteristics. As shown in Figure 8, the distribution of decreased *z*-values in VMHC showed an adjacent gradient declining trend among the groups. The lesion-side effect on VMHC following chronic pontine stroke was clearly observed, and the neural mechanisms related to this effect should be further discussed. The change in structures of fiber tracts, the gray matter volume, and the cortical pathways tends to be more obvious in the right hemisphere than the left hemisphere (Chiang et al., 2015; Liu et al., 2015, 2018; Diao et al., 2017). Generally, the more severe the structural damage, the more extensive the brain reorganization. As we discussed above, the change of VMHC is closely related to structural damage. Thus, this is possibly a neural mechanism underlying that the RP group shows a greater contribution to the VMHC than the LP group. In addition, all patients were right-handed, and previous studies have inferred that an increase in physical activity in daily life can result in increased activation of functional areas to compensate for the damaged ipsilesional area (Verstynen et al., 2005; Diao et al., 2017), specifically in right-handed patients with the left-dominant hemisphere stroke (Wang et al., 2019a). This phenomenon may suggest that the LP group will present with less damage in functional connectivity as a result of the compensation mechanisms compared to the RP group. Of course, further studies are needed to clarify the neural mechanisms underlying the lesion-side effect after chronic pontine stroke.

In the current study, the decreased bilateral lingual gyrus may predict declined performance of verbal working memory. As we know, the lingual gyrus is located in the ventral occipitotemporal cortex, the region that is responsible for word recognition and information integration to and from the language network (Lerma-Usabiaga et al., 2018; Ludersdorfer et al., 2019). Additionally, a previous study reported that memory deficits are closely related to the inferior occipital gyrus and the lingual gyrus (Kraft et al., 2014). Taken together, our results are in line with studies showing that the lingual gyrus in both hemispheres plays an important role in semantic processing, object priming, and output memory (Heath et al., 2012; Ding et al., 2015). Notably, a previous study on acute lacunar stroke reported increased VMHC in the lingual gyrus (Yang et al., 2017), which seems to contradict the current finding. The underlying mechanisms of such discrepancies remain unknown. One possible explanation is that the patient groups in different studies might reside in different disease stages. Furthermore, this is not contradictory to the decreasing trend of the lingual gyrus.

In contrast to both the left and right lingual gyrus, the precuneus was decreased only in the left hemisphere of the brain compared with HCs. However, in the *post-hoc* multiple comparison test, we found that this decreased result was most prominent in patients with RP. The disruption of homotopic FC is considered to be associated with structural impairment. Specifically, previous studies have demonstrated that patients with damage on the right side of the brain have significantly decreased FA values in the right pathway (Grieve et al., 2007; Liu et al., 2018). The unilateral reduced white matter integrity led to a decreased contralateral homotopic connectivity, and this might be a reasonable explanation for the change of VMHC in the Precuneus_L. Additionally, the alternation of the precuneus was consistent with the previously reported VMHC results and was supported by a longitudinal study (Shan et al., 2018). In our study, decreased Precuneus_L suggested a decline in the processing of working memory, including both the verbal working memory (VSTM and VLTM) and the visual working memory (Flanker task and spatial memory task). The default-mode network (DMN) is a collection of brain regions that are typically deactivated in goal-directed tasks and activated during rest periods. The more the default-mode activity of a subject correlated with the rest of the periods, the greater the activation of that subject to the visual and auditory stimuli (Greicius and Menon, 2004). As the main node in the DMN, the precuneus plays a central role in cognitive function and neural correlates of a functional connection between these regions, which may be associated with self-referential processing, attentional control, and working memory (Broyd et al., 2009; Liu et al., 2017). The damage to network hubs determines the potential for cognitive recovery after stroke (Aben et al., 2019; Wang et al., 2019b), so the Precuneus_L in the right hemisphere seems to be a great neurological biomarker for stroke-induced cognitive impairment.

In this study, the ACC of the spatial memory task showed the difference among the groups, and we have learned that the RP group played a critical role in our results, so we infer that the right-side stroke may be the main cause of more severe cognitive impairment and poor performance in spatial memory tasks with patients with chronic pontine stroke. To our knowledge, this study is not the first time to indicate that the right hemisphere plays a superior role in spatial tasks (Corbetta and Shulman, 2011; Liu et al., 2018; Shimonaga et al., 2020). In general, attention is considered to be the foundation of cognitive functions for the processing speed, the working memory, and the visuospatial processing we measured in the spatial memory task (Gajewski et al., 2018). The directed attention is thought to be related to spatial neglect, and the sustained attention is thought to be associated with cognitive processing speed, as evaluated by RT (Shimonaga et al., 2020, Lundqvist et al., 1997). The right hemisphere is proved to be responsible for maintaining a balance of attention between the two hemispheres (Fisk et al., 2002; Corbetta and Shulman, 2011). When there is damage to the right hemisphere, which is dominant in arousal, orientation, and duration, the result is a lack of precision to detect targets, leading to an increased RT (Lundqvist et al., 1997; Yuan et al., 2012; Ptak et al., 2020; Shimonaga et al., 2020). Moreover, mapping of the underlying neural mechanisms of the visuospatial working memory has been shown to consistently elicit activity in the right hemisphere of the dominant frontoparietal networks (Cheng et al., 2014; Lamp et al., 2016). Anyhow, our findings are congruent with the fact that the right hemisphere is more important to the change of cognitive function after stroke.

Nevertheless, the current results have facilitated a further understanding of cognitive function after pontine stroke. We encourage testing the modulation of these homotopic connectivities as potential targets for therapeutic intervention in the early phase of the stroke. By stimulating and enhancing such a target region of homotopic connectivity, we hope that this contributes to the effects of cognitive intervention trials, such as transcranial direct-current stimulation (Yun et al., 2015), which is crucial for the overall success of post-stroke rehabilitation.

## Conclusions

In this study, we explored the VMHC changes after chronic pontine stroke among the HC, LP, and RP groups, and compared each of the two groups using the *post-hoc* multiple comparison test. In addition, we demonstrated the relationships of decreased homotopic connectivity with cognitive impairment. Findings highlight the critical role of the lingual gyrus in the language network and the verbal working memory and of the value of the precuneus in working memory, attention control, and executive ability. Importantly, there was a lesion-side effect in decreased VMHC after chronic pontine stroke between the LP and RP groups. The RP group had a greater influence on the VMHC change, which is associated with cognitive function impairment in the cognitive task in this study; therefore, the right hemisphere is regarded as more vulnerable in cognitive impairment. Finally, we hope our findings may contribute to the improvement of cognitive performance after pontine stroke in the future.

## Limitations

Although interesting results and speculations have been discussed above, there are still several limitations in this study. First, as discussed above, the contribution of the left and right lesions varies between recruited patients, and more standardized larger samples need to be studied. Additionally, although we have learned that cognitive impairment exists in chronic stroke, there is little information about subcortical stroke and how this may impair the development along with the progression of stroke following onset. Therefore, a cross-sectional experimental design, or a longitudinal study design, should be applied in future studies. Finally, since a lesion-side effect on cognitive impairment after chronic pontine stroke was identified, multimodal MRI studies are required to clarify the underlying neural mechanisms. We hope that our results provide a basis for the development of a comprehensive and systematic understanding of post-stroke cognitive changes.

## Data Availability Statement

The original contributions presented in the study are included in the article/supplementary material, further inquiries can be directed to the corresponding author/s.

## Ethics Statement

The studies involving human participants were reviewed and approved by The First Affiliated Hospital of Zhengzhou University, Tianjin Medical University General Hospital and Tianjin Huanhu Hospital. The patients/participants provided their written informed consent to participate in this study.

## Author Contributions

All authors have made significant scientific contributions to this manuscript. LW, CW, YWe, PM, YWa, and JC conceived and designed the experiments. LW, CW, JL, JG, YWe, PM, and YWa performed the experiments. LW, YWe, and CW analyzed the data. LW, YWe, CW, and KW participated in the completion of the manuscript. All authors reviewed the manuscript.

## Conflict of Interest

KW is an employee of GE Healthcare. The remaining authors declare that the research was conducted in the absence of any commercial or financial relationships that could be construed as a potential conflict of interest.
